# Percutaneous Transvenous Angioplasty of Left Innominate Vein Stenosis Following Right Side Permanent Pacemaker Implantation- A Left Femoral Vein to Left Axillary Vein Approach

**DOI:** 10.1016/s0972-6292(16)30566-6

**Published:** 2012-12-02

**Authors:** Gautam Sharma, Nagendra Boopathy Senguttuvan, Sandeep Singh, Rajnish Juneja, Vinay K Bahl

**Affiliations:** Department of Cardiology, Cardiothoracic Sciences Centre, All India Institute of Medical Sciences, New Delhi, India

**Keywords:** Percutaneous Transvenous Angioplasty, Innominate Vein Stenosis, Permanent Pacemaker Implantation

## Abstract

Central venous stenosis after the insertion of a permanent pacemaker is a well recognized complication. This late complication is encountered when there is a need to change the pacemaker lead or extract it. We describe a young male who had such a complication after many years after right side pacemaker implantation. The lesion was managed percutaneously leading to placement of a new lead from the left side.

## Introduction

Central venous stenosis after the insertion of a permanent pacemaker is a well recognized complication. This late complication is encountered when there is a need to change the pacemaker lead or extract it. Various treatment modalities like lead extraction, venous channel dilatation and surgical bypass have been adopted to manage it. We describe one such patient with a central vein stenosis, following permanent pacemaker implantation, who was successfully managed using coronary intervention hardware in an innovative fashion.

## Case description

**Figure 1:**
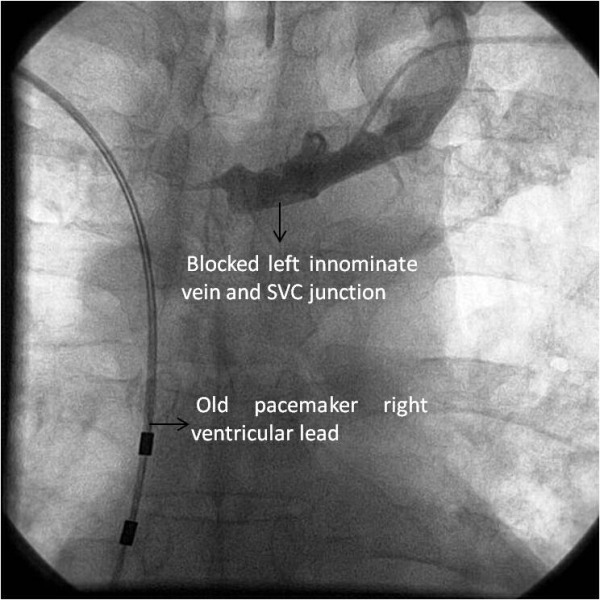
Contrast injection into left subclavian showed blocked left brachiocephalic - superior venacava junction

**Figure 2:**
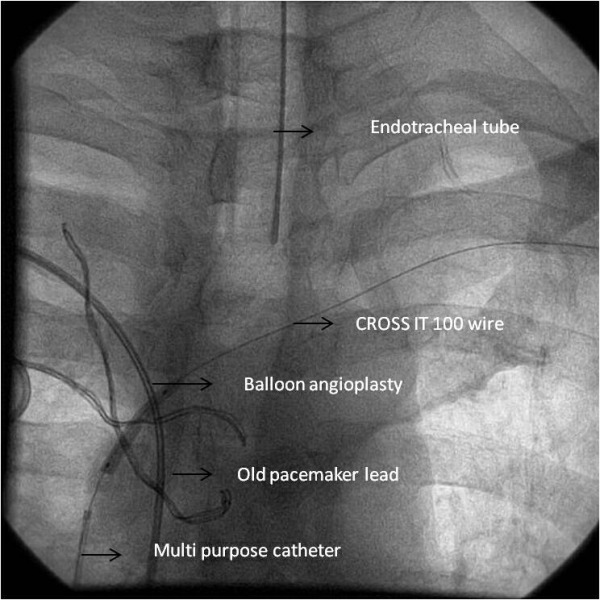
Balloon angioplasty of the junction of superior venacava and left brachiocephalic vein over the wire that has been snared out through the left axillary vein.

A 38 year old male patient with a single chamber pacemaker for symptomatic congenital complete heart block via the right subclavian vein approach a decade back, presented with pre-syncope that was determined to be because of intermittent failure to capture. Pacemaker interrogation revealed preserved battery voltage, high lead impedance and failure to capture at maximum output. Immediate temporary pacemaker was inserted through the right femoral vein. Baseline investigations were unremarkable and echocardiography done showed a normal left ventricular function. The patient was taken up for the lead replacement. Right axillary vein was successfully accessed but the guide wire could not be negotiated beyond a short distance. A venogram through the puncture needle revealed a blocked right innominate vein. We then proceeded to attempt from the left side. Axillary vein access was achieved successfully but the guidewire could not be negotiated beyond a short distance similar to what we had encountered in the right side. An angiogram of the left sided venous system through the partially inserted sheath was done which revealed an occluded left innominate venous system (Figure 1 and Video 1). With a multipurpose catheter introduced through the left femoral vein, a venogram at the level of superior vencava (SVC) was done. It confirmed the occlusion at the junction of SVC and bilateral brachiocephalic veins (Video 2). Lead extraction tools could have been utilized to remove the old lead and at the same time create a path to place a new lead. But financial constraints and limited experience made us to consider other options. We, after deliberation, proceeded to attempt transvenous angioplasty of SVC and left innominate vein. We used the multipurpose catheter that was positioned at the precise site of occlusion, from below in the same fashion as a coronary guiding catheter. The position of the guiding catheter was confirmed to be at the stenotic site with contrast and a 014" balanced middle weight (BMW)(Abbott Laboratories, IL,USA) guide wire was initially used to cross the "chronic total venous occlusion". Since we were not able to cross the lesion with it, a stiffer CROSS IT 100XT wire (Abbott Laboratories, IL,USA) was utilized to cross the lesion. The end of the wire now in the lumen of the left innominate vein, was snared through the left axillary vein and exteriorized (Video 3). A Voyager 3.5x20 mm balloon (Abbott Laboratories, IL,USA) was passed over the exteriorised end of the wire. Multiple dilatations were given at the site of block (Figure 2). A 035" Glidewire (Terumo Corp, NJ, USA) was negotiated through the now dilated lesion from the axillary vein to the inferior venacava (IVC). Graded dilatations were given to the lesion with an 8F sheath and a 9 F sheath over the glide wire. Finally, customized coronary sinus sheath was passed into the RA via the left axillary vein through which the right ventricular pacing lead was placed. Post procedure, the patient had an uneventful course. He was shortly discharged with good pacing parameters.

## Discussion

Incidence of central venous stenosis in a large study was found to be 26% with 9% of patients having total obstruction and 17% having partial obstruction [1]. In spite of long experience with transcutaneously implanted pacing systems, the risk factors for the development of venous stenosis are not clear. While presence of multiple pacemaker leads, oestrogen therapy, history of venous thrombosis, usage of temporary wire before implantation and infections have been described as possible risk factors associated with increased risk of stenosis, use of anticoagulation had been found to have a protective influence. Though most of them are asymptomatic, some may present with obstructive symptoms. The pathogenesis of central venous stenosis includes acute thrombosis and infection. Acute thrombosis that might be due to endothelial damage by puncture or the shearing effect of the lead is followed by fibrosis and stenosis. Bracke et al [2] found that infection of pacemaker was associated with fivefold increased risk of venous occlusion. The most common site of stenosis is the junction of the innominate and superior vena cava. Our patient had a pacing lead on one side but he had developed a stenosis on the contralateral side also which might be related to the spread of inflammation to the other side. His being a chronic smoker might have also contributed to the thrombotic occlusion. Venous occlusion creates problems during redo procedures for lead replacement that have been tackled by various methods. Kastner et. al [3] have reported a successful venoplasty in SVC syndrome caused by pacing leads, and showed patency of these veins at 6 months. Lead extraction and SVC angioplasty was done in three patients with SVC obstruction following pacemaker lead implanation with good intermediate term follow-up [4]. Tourret et al [5] described successful angioplasty via the cervical veins of a patient with venous stenosis following a pacemaker and an ipsilateral hemodialysis fistula. These authors also emphasized that patients with renal dysfunction who needed a pacemaker are at higher risk of developing venous stenosis. Various venous approaches have been utilized for such therapy. Successful pacemaker implantation following angioplasty via the subclavian approach was described in a patient who developed cervical venous stenosis many years following a lead extraction [6]. Groin approach has been utilized in three patients with pacemaker lead induced SVC syndrome by angioplasty followed by stent insertion [7]. This report confirmed the safety of stents in this situation, since there were concerns regarding impingement of lead by the stent. Finally, Kolb et al [8] described insertion of a very thin bipolar pacing lead despite the presence of tight venous stenosis.

Our case illustrates two interesting aspects: 1) Occurrence of bilateral innominate vein stenosis after pacemaker insertion on one side and 2) a novel approach to pacemaker lead insertion, when both upperlimb veins are occluded. In the absence of availability of lead extraction hardware or expertise, this unique transfemoral approach for percutaneous trans-luminal balloon venoplasty of innominate veins followed by lead insertion through the axillary vein is a safe and a feasible option.

## Figures and Tables

**Video 1:** Contrast injection into left brachiocephalic vein showed blocked junction of left brachiocephalic vein and SVC : Click here to view the movie

**Video 2:** Contrast injection into Superior Venacava (SVC) showed blocked junction of bilateral innominate vein and SVC: Click here to view the movie

**Video 3:** Guidewire was introduced from the left femoral vein. Entire course of the wire was left femoral vein > inferior vena cava > right atrium > superior vena cava > left innominate vein > left subclavian vein > exteriorized through left axillary vein. Click here to view the movie

## References

[R1] Lickfett L (2004). Incidence of venous obstruction following insertion of an implantable cardioverter deﬁbrillator. A study of systematic contrast venography on patients presenting for their ﬁrst elective ICD generator replacement. Europace.

[R2] Bracke F (2003). Venous occlusion of the access vein in patients referred for lead extraction: influence of patient and lead characteristics. Pacing Clin Electrophysiol.

[R3] Kastner RJ (1996). Pacemaker-induced superior vena cava syndrome with successful treatment by balloon venoplasty. Am. J. Cardiol.

[R4] Bolad I (2005). Percutaneous treatment of superior vena cava obstruction following transvenous device implantation. Catheter Cardiovasc Interv.

[R5] Tourret J (2005). Central venous stenosis as a complication of ipsilateral haemodialysis fistula and pacemaker. Nephrol. Dial. Transplant.

[R6] Kim JS (2005). A Case of Pacemaker Implantation after Balloon Venoplasty on Innominate Vein Stenosis. Korean Circulation Journal.

[R7] Teo N (2002). Treatment of superior vena cava obstruction secondary to pacemaker wires with balloon venoplasty and insertion of metallic stents. Eur. Heart J.

[R8] Kolb C (2005). Successful pacemaker lead implantation via the subclavian vein despite of high degree stenosis of the vein. Pacing Clin Electrophysiol.

